# *ι*-Carrageenan Manganese Oxide Bionanocomposites as a Promising Solution to Agricultural Challenges

**DOI:** 10.3390/ma18030495

**Published:** 2025-01-22

**Authors:** Spartak S. Khutsishvili, Nino Gagelidze, Astghik S. Tsokolakyan, Mkrtich A. Yeranosyan, Eteri Tkesheliadze, Vardan A. Sargsyan, Darejan Dughashvili, Natela Dzebisashvili, Keso Aronia, Archil Benashvili, Dali Dzanashvili, Irine Gurgenidze, Grigor Tatishvili, Paula Fraga-García

**Affiliations:** 1 Rafael Agladze Institute of Inorganic Chemistry and Electrochemistry, Ivane Javakhishvili Tbilisi State University, 11 Mindeli Str., 0186 Tbilisi, Georgia; d_dughashvili@yahoo.com (D.D.); n.dvalishvili@gtu.ge (N.D.); achi_ben@yahoo.com (A.B.); dalidzanashvili@tsu.ge (D.D.); irina_gurgenidze@yahoo.com (I.G.); tati@iice.ge (G.T.); 2 Agricultural University of Georgia, Sergi Durmishidze Institute of Biochemistry and Biotechnology, Kakha Bendukidze University Campus, 240 D. Aghmashenebeli Al., 0159 Tbilisi, Georgia; n.gagelidze@agruni.edu.ge (N.G.); etkes2015@agruni.edu.ge (E.T.); 3 Innovation Center for Nanoscience and Technologies, A.B. Nalbandyan Institute of Chemical Physics NAS RA, 5/2 P. Sevak Str., Yerevan 0014, Armenia; astghik.tsokolakyan@edu.isec.am (A.S.T.); myeranos@ysu.am (M.A.Y.); 4 Department of Organic Chemistry, Yerevan State University, 1 A. Manukyan Str., Yerevan 0025, Armenia; vardan.sargsyan1@ysu.am; 5 Institute of Hydrometeorology, Georgian Technical University, 150g D. Aghmashenebeli Ave., 0112 Tbilisi, Georgia; 6 Department of Chemistry, Sokhumi State University, 61 Politkovskaya Str., 0186 Tbilisi, Georgia; karonia18@gmail.com; 7 Chair of Bioseparation Engineering, Department of Energy and Process Engineering, School of Engineering and Design, Technical University of Munich, 85748 Garching, Germany; p.fraga@tum.de

**Keywords:** hybrid nanoparticle characterization, natural polysaccharide shell, carbohydrate nanocomposite, sulfated antimicrobial bionanocomposite, *Clavibacter sepedonicus*

## Abstract

Agriculture faces numerous challenges: infectious diseases through phytopathogens and soil nutrient deficiencies hinder plant growth, reducing crop yields. Biopolymer nanocomposites offer promising solutions to these challenges. In this work, we synthesize and characterize novel bionanocomposites (*ι*-CG-Mn) of manganese (hydr)oxide nanoparticles (approx. 3 to 11 nm) embedded in the matrix of the natural polysaccharide *ι*-carrageenan (*ι*-CG). Using spectroscopic methods we verify the presence of the nanoparticles in the polymer matrix while leaving the polysaccharide structural characteristics unaffected. Elemental analysis determines the mass content of metal ions in the *ι*-CG-Mn to be approx. 1 wt%. Electron microscopy techniques show the supramolecular organization of the *ι*-CG-Mn and the homogeneous nanoparticle distribution in the polymer matrix, while thermal analysis reveals that the bionanocomposite maintains high thermal stability. Moreover, the co-incubation of the phytopathogen *Clavibacter sepedonicus* with *ι*-CG-Mn inhibits the pathogen growth by 67% compared to the control. Our bionanocomposites demonstrate (1) strong bactericidal activity and (2) potential as microfertilizers that stimulate agricultural plant growth through the dosage of metal ions. These properties arise from the bioactivity of the widely available, naturally sulfated polysaccharide biopolymer matrix, combined with the antimicrobial effects of manganese (hydr)oxide nanoparticles, which together enhance the efficacy of the biocomposite. The non-toxic, biocompatible, and biodegradable nature of this biopolymer satisfies the high environmental demands for future biotechnological and agricultural technologies.

## 1. Introduction

Polymer metal-containing nanocomposites have already demonstrated excellent performance in biomedical applications [1,2,3]. The success of polymer composites is ensured by the synergy of the properties of polymer matrices and the unique properties of metal-containing nanoparticles. Organic high-molecular compounds act as a reliable stabilizing system in the formation of nanoparticles through Coulomb, coordination, van der Waals, and other interactions with the surface of nanoparticles [4]. Biopolymers are important as stabilizing matrices, which have highly polar hydroxyl, as well as carbonyl and carboxyl groups of polysaccharides. Natural polysaccharides have valuable properties, such as biocompatibility, non-toxicity, biodegradability, hydrogelation, hydrotropism (the property of increasing the solubility of poorly soluble substances), and many others [5]. These properties have given a new impetus to the development of modern nanomaterials for agriculture [6,7,8]. In addition to the obvious use of nanomaterials to enrich soil with minerals, polysaccharide-based nanosubstances can promote rapid seed germination, protect plants from various diseases, increase their resistance to different stresses, help retain and transport water in plant tissues, as well as increase crop yields, etc.

Materials containing manganese are gaining attention for the creation of new nanosubstances based on high-spin metal ions for biomedicine [9]. In particular, manganese oxide nanoparticles exhibit antioxidant [10] and antibacterial properties [10,11], both very valuable characteristics for the creation of new materials for agricultural purposes. The importance of manganese itself in the biochemical processes of plants is difficult to overstate [12,13]. This metal ion is a cofactor of many plant cell enzymes involved in the redox reactions of photosynthesis, and the synthesis of vitamins C, B, E, and ascorbic acid. In addition, manganese increases the content of sugars and their outflow from the leaves; manganese also accelerates plant growth and seed ripening. At the same time, a deficiency of manganese slows the synthesis of organic substances, leading to a decrease in the amount of chlorophyll, thereby inducing chlorosis in spots of leaves [14,15,16]. Manganese-containing nanocomposites based on polysaccharides are capable of gradually degrading and supplying plants with the necessary element in the required and safe doses in various types of soil, and simultaneously prevent the leaching of manganese from the soil.

In our previous work, we characterized manganese-containing bionanocomposites based on a series of polysaccharides (arabinogalactan, sulfated arabinogalactan, and κ-carrageenan) and studied their application as a new form of microfertilizer [12]. Among the biopolymer matrices employed in the creation of multifunctional nanomaterials for agriculture, sulfated polysaccharides have turned out to be the most promising. These nanocomposites have a better stimulating effect on potatoes during the growing season, reduce the level of plant stress, and show more pronounced antibacterial activity. However, arabinogalactan is expensive and not very widely available; therefore, we have decided to focus here on another naturally sulfated polysaccharide, carrageenans. In this paper we use *ι*-CG to design novel multifunctional manganese-containing nanomaterials for agricultural purposes. *ι*-CG is a natural sulfated polysaccharide and is mainly produced from the red alga *Eucheuma denticulatum* [17]. Structurally, carrageenans are polysaccharides of alternating subunits of 3-linked *β*-D-galactopyranose and 4-linked *α*-D-galactopyranose or 4-linked 3,6-anhydro-*α*-D-galactopyranose, forming the disaccharide repeating unit of carrageenans [18,19]. The most important difference affecting the properties of the different classes of carrageenans is the number and position of sulfate esters on the repeating galactose subunits. Unlike *κ*-carrageenan, in the *ι*-CG each galactose molecule has a sulfate group. The presence in the structure of the *ι*-CG of a highly ionizable sulfate group imparts additional polycationic and polyanionic properties. The high amount of sulfate esters lowers the dissolution temperature of carrageenans, compared to other polysacchardies, and leads to the formation of softer gels, an advantageous property, which explains why they are often used in biomedicine and the food industry as a stabilizing and thickening agent [20].

The manuscript presents the results of the synthesis of novel bionanocomposite *ι*-CG-Mn based on the naturally sulfated polysaccharide *ι*-CG with manganese (hydr)oxide nanoparticles. We study their activity as potential microfertilizers with advantages for agricultural applications. They stimulate plant development, increase plant stress resistance, and possess bactericidal properties against phytopathogens [12,21]. The phytopathogen bacterium *Clavibacter michiganensis* subsp. *sepedonicus* (*Cms*) is a quarantine object in most countries of the world and is the cause of serious crop losses (up to half of the annual losses in potato production originate from *Cms*) [22,23]. Hence, one essential task in agricultural research is to obtain effective nanocomposites that exhibit antimicrobial impact and suppress the viability of such phytopathogens. Therefore, in an attempt to create more effective bionanocomposites, this work comprehensively examines the structural features and physicochemical properties of the resulting *ι*-CG-Mn nanosystem, and its bactericidal activity against *Cms*. Our investigations are critically important for developing modern plant growth methods as well as agriculture based on safe and biodegradable materials. Our goal is to produce healthy plant material free from a number of phytopathogens.

## 2. Materials and Methods

### 2.1. Synthesis of Manganese-Containing Nanocomposites

The *ι*-CG was purchased from Sigma-Aldrich (Darmstadt, Germany). Nanobiocomposite *ι*-CG-Mn was synthesized according to the following procedures [24]: *ι*-CG (2 g) was kept under stirring in H_2_O (100 mL) upon heating at 50 °C to a homogeneous medium. Then, MnSO_4_ × 5H_2_O (0.46 g) in H_2_O (2 mL) and NH_4_OH (0.2 mL) were added. After 24 h, the reaction product was precipitated into alcohol and dried. Thorough washing of the precipitate with alcohol and drying gave 1.63 g of *ι*-CG-Mn nanocomposite, with a yield of 81%.

The resulting nanocomposite was a light-brown powder. Usually, the color intensity depends on the mass content of manganese in the sample. The average mass of manganese in *ι*-CG-Mn is 0.7–1.5 wt%. The *ι*-CG-Mn nanocomposite is water-soluble through swelling and capable of forming gels.

### 2.2. Physicochemical Measurements

Elemental analysis of the original *ι*-CG and obtained *ι*-CG-Mn was performed on a Skyray Instruments EDX3600H X-ray fluorescence spectrometer (Dallas, TX, USA). Additionally, the percentage of manganese in the *ι*-CG-Mn was determined using the energy-dispersive X-ray spectroscopy function built into a SEM TM3030 Plus (Hitachi, Tokyo, Japan) chamber. Here, the atoms of the sample are excited via electron beam, and, thus, emitted X-rays of wavelengths characteristic of each chemical element. Analyzing the energy spectrum of X-ray emissions, we assessed the sample qualitative and quantitative composition. The number of repetitions for each sample was five, as well as five measurement areas in each sample. The elemental analysis results are presented in Section 2.1 and 3.1.

The TG-DSC study was carried out using a NETSCH STA 2500 Regulus (Selb, Germany) synchronous thermal analysis device. The weighed samples (in the form of powders) were heated from 20 to 800 °C at a rate of 10 °C per minute. The studies were carried out in an oxidizing (air) atmosphere in a corundum crucible for DSC with a height of 6 mm and diameter of 3 mm. The amount of air was sufficient for the complete oxidation of the test samples.

The UV-vis spectra were recorded on a Cary 60 UV-Vis spectrophotometer (Santa Clara, CA, USA). For obtaining the UV-vis spectra of the samples, 1 mg/mL of each *ι*-CG and *ι*-CG-Mn solid samples were dissolved in deionized water and characterized with UV-vis spectroscopy.

The FTIR spectra were recorded on a Spectrum Two Perkin Elmer FT-IR spectrometer (Shelton, CT, USA). The Raman spectra were recorded using a LabRAM HR Evolution HORIBA Raman spectrometer (HORIBA France SAS, Lyon, France).

The EPR spectra were recorded on a Bruker (Billerica, MA, USA) EMXplus spectrometer (X-band 9.88 GHz). CW EPR spectra were recorded under the following conditions (in quartz ampoules with a diameter of 3 mm): modulation amplitude 10 G, frequency modulation 100 kHz, time constant 327.68 ms, conversion time 262.14 ms, microwave power 0.6325 mW, average number of scans 1–5, amplification 60 dB, Q value 7200 at room temperature. The spin concentration of the nanocomposite *ι*-CG-Mn was estimated using the spectrometer Xenon 1.2 software.

To study the morphology of the *ι*-CG-Mn film, the obtained nanocomposite was dispersed in double distilled water (dd-water) at concentrations 0.01–0.1 g/L [25]. The sample for TEM was then dropped onto a copper grid that had previously been plasma-activated and air-dried [25]. The prepared sample was examined using a JEM-1400 Plus TEM device (JEOL, Tokyo, Japan) at an accelerating voltage of 80 kV. For additional TEM analyses, 10 µL of the samples were left to adhere for 10 min on glow-discharged 200 mesh grids with a carbon-enhanced formvar film. After that, the excess liquid was either blotted with a filter paper, or the grid was first washed three times on drops of dd-water before blotting. The fully dried grid was then imaged in a JEM-1400 (JEOL, Tokyo, Japan) at 120 kV using the Veleta 2k CCD Olympus camera (Tokyo, Japan).

### 2.3. Bacterial Culture

*Cms* is a short, non-motile, Gram-positive, rod-shaped bacterium. The pathogen is aerobic, but slow growth can be observed in anaerobic conditions. Colonies are creamy and yellowish. The optimum growth temperature is 20–23 °C. Gram-stained cells can appear in an L- or V-formations [26].

**Figure 1:**
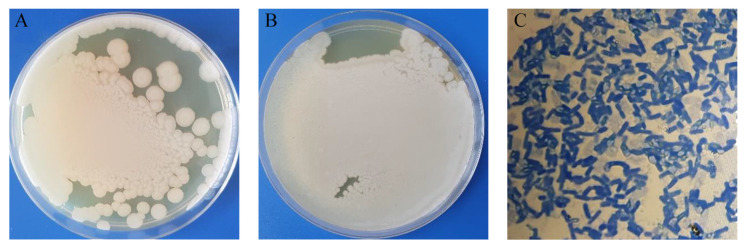
Obtaining of pure cultures of (**A**) *Cms* NCPPB 2137, (**B**) the bacterial lawn culture on the surface of GPY agar Petri dish, (**C**) and Gram-stained culture.

The strain of *Cms* NCPPB 2137, a causative agent of potato ring rot, was derived from the National Collection of Plant Pathogenic Bacteria (bacterial culture repository hosted and maintained by Fera Science (York, UK), see Figure 1.

*K. pneumoniae* is a Gram-negative, non-motile, encapsulated, lactose-fermenting, facultative anaerobic, rod-shaped bacterium. It appears as a mucoid lactose fermenter on MacConkey agar. *E. coli* is a Gram-negative, facultative anaerobic, rod-shaped, coliform bacterium of the genus *Escherichia*. The strains *K. pneumoniae* ATCC 13833 and *E. coli* ATCC 25922 were derived from the American Type Culture Collection (Manassas, VA, USA).

The strain was resuscitated in tryptic soy broth (TSB). To obtain pure colonies, the inoculum was transferred to Petri dishes with tryptic soy agar (TSA) and placed in a thermostat at 25 °C for 48 h. For further experiments, the strain was cultured on GPY nutrient medium (g/L): yeast extract 5, glucose 5, peptone 10, NaCl 5, and agar 17, sterilized at 121 °C for 20 min [27]. The cultures are maintained on slant agar in a thermostat at 25 °C.

### 2.4. Bactericidal and Bacteriostatic Effect of ι-CG-Mn

In order to determine the bacteriostatic activity of the nanocomposite, two methods were used in the study:

The agar diffusion method was used to investigate the bactericidal properties of the nanocomposite *ι*-CG-Mn [28]. For the experiment, a pure bacterial culture from the solid medium was transferred to GPY liquid medium and placed on an incubating shaker at 80 rpm, at 25 °C in the dark for 48 h. The bacterial suspension was inoculated onto GPY agar through a sterile wood cotton swab. After 10 min, wells were made on the agar and added with the aqueous solution of *ι*-CG-Mn and the original natural polysaccharide *ι*-CG at various concentrations, as well as sterile water in the control wells. The effective choice of concentrations was made based on the concentrations used for nanocomposites based on polysaccharides, in previous experiments [7,12,29,30]. Inhibition of bacterial growth was measured by the diameter of the clear zone around the block.

The optical turbidity (D_595_ nm) of the bacterial suspension was used to determine the bacteriostatic activity [31]. Therefore, we used an AE-S70-2U Series UV-VIS spectrophotometer (AELAB, Guangzhou, China). In the control variant, a 48 h inoculum of *Cms* in the amount of 3% was added to the above-mentioned liquid nutrient medium (100 mL), whereas in the experimental variant, a 3% inoculum and 1% *ι*-CG-Mn (3.93 g/L) were introduced. Every two hours, the turbidity of both samples, the control and the *ι*-CG-Mn dotted samples, was measured using a spectrophotometer.

Statistical data processing was carried out using the SigmaPlot v.12.5 program (SYSTAT Software, Chicago, IL, USA). The data obtained after treatment were compared statistically with controls using the nonparametric Mann–Whitney U test.

## 3. Results

### 3.1. Synthesis and Spectroscopic Characterization of the ι-CG-Mn Bionanocomposites

The structural organization of *ι*-CG is in alternating subunits of two galactose molecules, with a sulfate group on each galactose molecule [18,20]. The *ι*-CG macromolecules themselves are very large, flexible, and capable of forming twisted structures and can also form soft gels at room temperature. In addition, carrageenans have a high tendency to form coordination biopolymer complexes with the metal ions located on the surface of metal ion-containing nanoparticles. In this way, *ι*-CG helps produce promising aggregation-stable composite materials, e.g., with manganese-containing nanoparticles. The resulting nanocomposite *ι*-CG-Mn was synthesized employing the co-precipitation method in the reaction of manganese sulfate MnSO_4_ × 5H_2_O with ammonium hydroxide in the presence of the polysaccharide *ι*-CG (see Section 2.1). The co-precipitation method offers various advantages in terms of simplicity, rapidity of preparation, and ease of particle size control [32]. Moreover, the alkaline synthesis environment plays a decisive role. In the basic aqueous medium, the carboxylate fragments are highly negatively charged leading to a change in the configuration of the carrageenan side chains from a coiled polymer structure to an uncoiled conformation due to the repulsion between the negative charges. These negatively charged sites become therefore sterically accessible and are able to interact electrostatically with the positively charged sites on the metal oxide nanoparticles, thus stabilizing the nanoparticles in solution [33,34]. The whole mixture was stirred during synthesis to ensure uniform distribution of manganese in the biopolymer and stabilization of nanoparticles in its mass, which was confirmed by a change in the color of the solution to brown. We carried out elemental and EDX analysis of the samples and obtained an average manganese mass value in the synthesized *ι*-CG-Mn of 0.7–1.5 wt%. It is most likely that manganese nanoparticles in the resulting nanocomposites are chemically notated as Mn(OH)_x_ × nH_2_O, stabilized in complexly organized high-molecular *ι*-CG fibers on oxygen-containing groups (primarily hydroxyl groups) similar to other polysaccharides [24,35]. However, various compositions of nanocrystallites of manganese oxides are possible, along with their polymorphic modifications [32,36], and without a stabilizing agent, the particles form larger aggregates, which demonstrates the advantage of our method.

**Figure 2:**
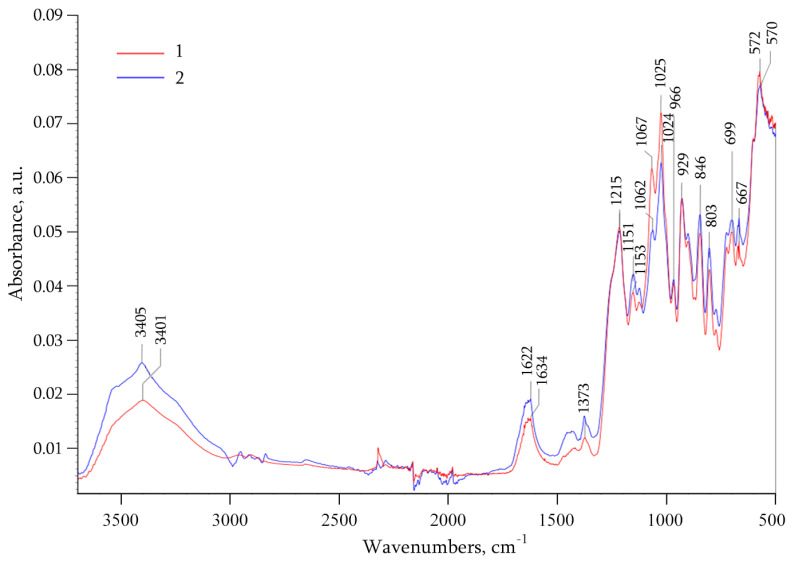
FTIR spectra of the original *ι*-CG (1) and the nanocomposite *ι*-CG-Mn (2).

Based on Fourier transform infrared (FTIR) spectroscopy, the nanocomposite forms without changing the structure of the original organic matrix, and the spectrum of *ι*-CG-Mn displays the characteristic peaks of *ι*-CG (Figure 2). Most of the peaks from the FTIR spectra of both *ι*-CG and *ι*-CG-Mn appear in the same positions, with no strong shifts observable among both spectra [37]. The main changes in *ι*-CG-Mn compared to the original *ι*-CG are noticeable in the high-frequency region: there is a broadening of the absorption region 3000–3600 cm^−1^, caused by stretching vibrations of hydroxyl groups *δ*(OH), indicating a complex formation with Mn^2+^ and the presence of bound water [38,39,40]. The peaks at 1633 cm^−1^ appear from polysaccharide-bound water, at 1215 cm^−1^ for asymmetric stretching of O=S=O (ester sulfate group) and at 1066 cm^−1^ for glycosidic bonds in carrageenan. The absorption bands at 930 cm^−1^ contribute to C–O–C of 3,6-anhydrogalactose and 845 cm^−1^ related to CO–O–SO_3_ vibrations at D-galactose-2-sulphate and D-galactose-4-sulphate. This establishes the existence of galactose in *ι*-CG and *ι*-CG-Mn. In addition, the increase in the shoulder peak at 700-710 cm^−1^ is due to the Mn–O vibration in the manganese oxide nanoparticles [41]. The main broadening of OH stretching vibrations in the spectra of the *ι*-CG-Mn during complexation is associated with the participation of OH groups in metal-ligand bonding and the presence of a strong hydrogen bond network between the OH groups of the *ι*-CG-Mn polysaccharide matrix and H_2_O molecules in the manganese-polysaccharide crystal structures. These interactions stabilize the nanocrystals in the biopolymer and prevent their further aggregation. Such a system of hydrogen bonds, which is also associated with ionization due to complexation, has been found in the crystal structure of the Mn(D-gluconate)_2_ × 2H_2_O salt; we also observed it in previous work with manganese-containing nanocomposites [12,42].

**Figure 3:**
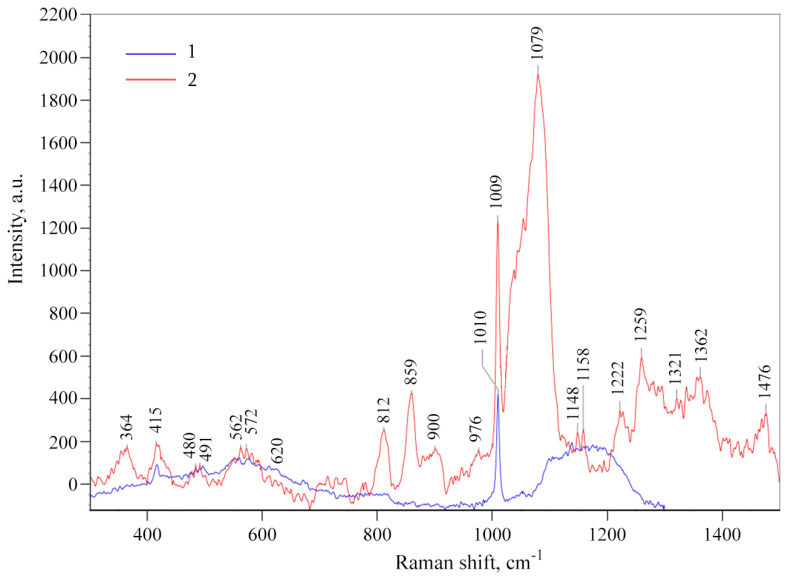
Raman spectra of the original *ι*-CG (1) and the nanocomposite *ι*-CG-Mn (2).

The original *ι*-CG and the *ι*-CG-Mn nanocomposite were also analyzed using Raman spectroscopy (Figure 3). The characteristic peaks for *ι*-CG are the band at 850 cm^−1^, 905–907 cm^−1^, 925–935 cm^−1^, 970–975 cm^−1^, 1075–1085 cm^−1^, and 1240–1260 cm^−1^ according to Pereira et al. [43]. In contrast to *ι*-CG-Mn, the spectrum of the pure *ι*-CG sample did not show pronounced signals, only the signal located at 1009 cm^−1^, which corresponds to the sulfate group characteristic for carrageenan. In the Raman spectra of the *ι*-CG-Mn, the peaks are observed at 859 cm^−1^, 1079 cm^−1^, and 1259 cm^−1^. The shift to higher frequencies of the peak appearing at 859 cm^−1^ from the 850 cm^−1^ reported in the literature corresponding to the position of CO–O–SO_3_ (on C-4 of galactose) [43,44] might indicate sulfate groups, helping to stabilize the manganese-containing nanoparticles. The formation of a metal complex with a heavy atom is usually accompanied by a shift of peaks to a higher wavenumber and sometimes by some increase in its intensity [45]. The main peak characteristics of manganese oxides are located in the 460–485 cm^−1^, 560–575 cm^−1^, and 615–660 cm^−1^ regions [46]; however, intense peaks for the *ι*-CG-Mn are not observed in the spectrum, which can be explained by the low mass fraction of manganese in the nanocomposite (range 0.7–1.5 wt%), the very small size of the nanocrystal units, and the formation of various polymorphic modifications of manganese oxides.

**Figure 4:**
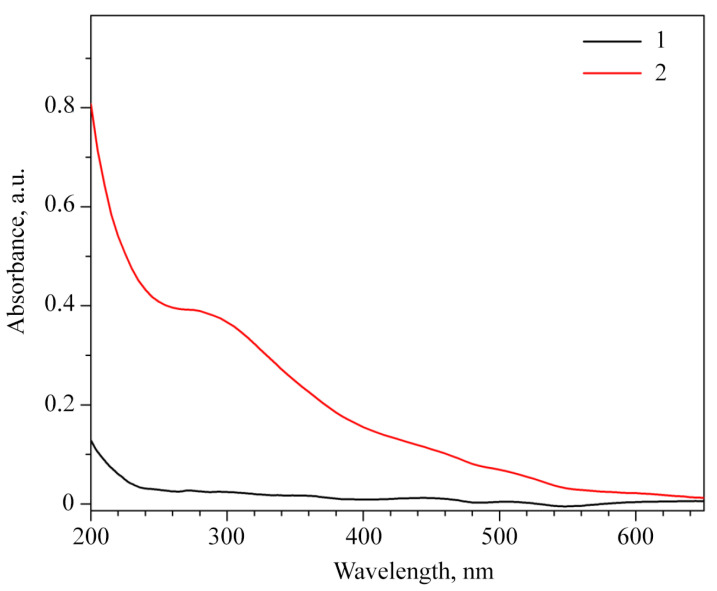
The UV-vis spectra of the original *ι*-CG (1) and the nanocomposite *ι*-CG-Mn (2).

In the Ultraviolet-visible (UV-vis) spectrum of *ι*-CG-Mn, there is an absorption band in the region of 280 nm, caused by MnO_2_ nanoparticles [41,47], see Figure 4. However, no such peak is observed for the original polysaccharide *ι*-CG. The literature reports absorption of manganese oxide in the range of 210–280 nm [48], which may be due to a transition between electron bands. According to data in another article, the absorption around 280 nm is due to n → π* or π → π* transitions of manganese oxides [49].

**Figure 5:**
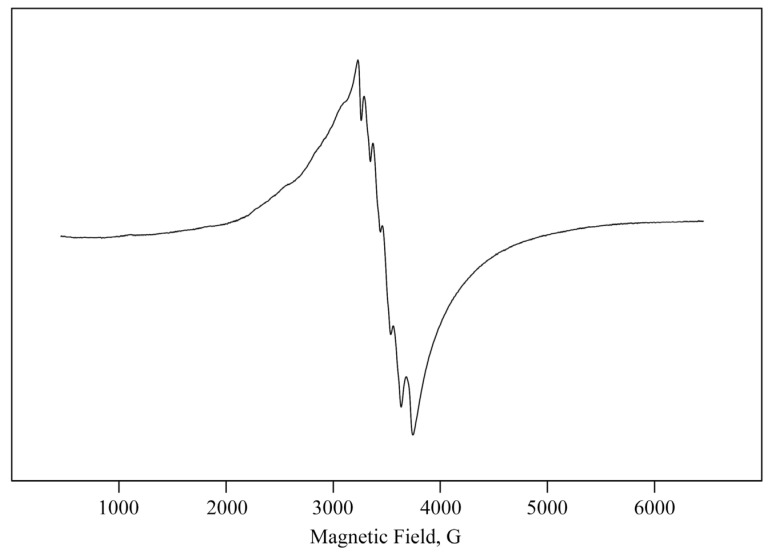
The EPR spectrum of the nanocomposite *ι*-CG-Mn.

The original polysaccharide *ι*-CG is diamagnetic, while the nanocomposite *ι*-CG-Mn is paramagnetic with a spin concentration of about 2.0 × 10^16^ spins/mm^3^. In the electron paramagnetic resonance (EPR) spectrum, intense complex lines are observed in the nanocomposite (Figure 5). The ground state of high-spin metal ions with the *d*^5^ configuration is an orbital singlet, and its spectrum contains six lines of Mn^2+^ hyperfine components (HFS) with spin 5/2 with the parameters of the splitting constant (*A*) 95.9 G and the average line width (Δ*H*) 45.9 G in the region of the *g*-factor 2.007(1) [50]. Also, in the EPR spectrum, a second broad almost isotropic Lorentzian line is observed (in almost ideal cubic symmetry [51]) with an average effective *g*-factor of 2.08(1), anisotropy parameter (*A*/*B*) 0.85, and a width peak-to-peak (Δ*H*) of 470–480 Gs. Such a broad line width is explained by the effect of exchange narrowing due to the close proximity of paramagnetic metal ions, which causes strong dipole and spin exchange interactions [52,53]. In our case, the close proximity of Mn^2+^ ions could have caused strong exchange interactions that may have resulted in a single-line EPR spectrum in the process of nanoparticle formation. As the content of Mn^2+^ increases during crystal growth, manganese-containing clusters are generated. These exchange interactions gradually degrade the resolution of the HFS components in the spectrum [54]. The deviation in the value of the symmetry parameter (*A*/*B*) from 1.0 in this case is probably due to the dispersion of the sizes of the resulting nanoparticles and their distribution in the biopolymer matrix.

### 3.2. Thermal Analysis of the Nanocomposite ι-CG-Mn

**Figure 6:**
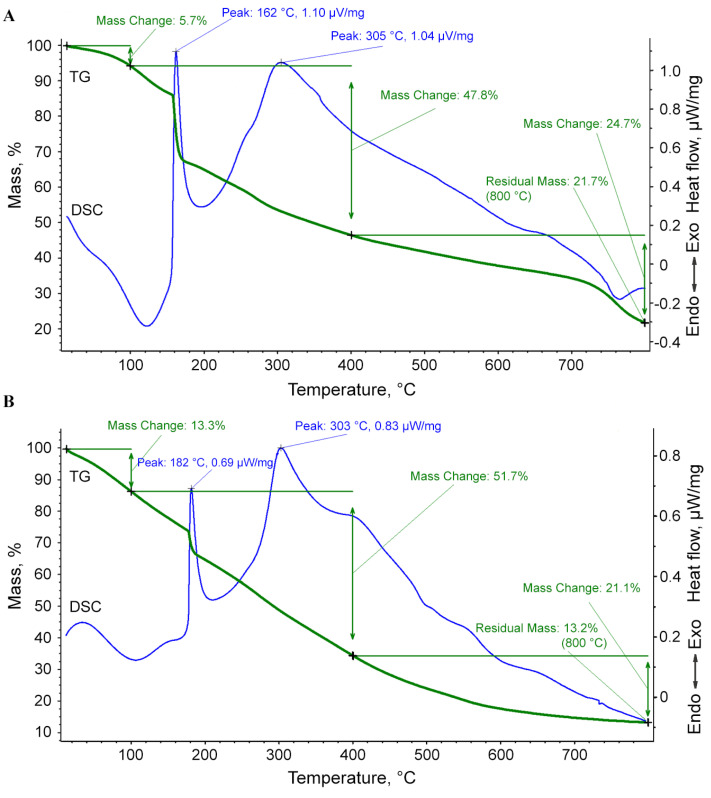
Curves of mass loss (green) and heat effects (blue) of the thermostability of the nanocomposite *ι*-CG-Mn (**A**) and the original *ι*-CG (**B**).

With further use of the nanocomposites, an important question will inevitably arise about the range of their thermal stability during heat treatment, which occurs, for example, during the sterilization of nanocomposites and their aqueous solutions by autoclaving under standard conditions in the manufacture of nutrient media for plant material. According to the results of simultaneous thermal analysis (differential scanning calorimetry (DSC) and thermogravimetric (TG) analysis) for the nanocomposite *ι*-CG-Mn in an oxidizing atmosphere up to 800 °C at a heating rate of 10 °C per minute, several thermal effects of different signs and magnitudes were revealed (Figure 6A). For a comparative analysis and determination of the effect of manganese (hydr)oxide nanoparticles on the polysaccharide matrix, we conducted an experiment with the original *ι*-CG as a reference (Figure 6B).

In the temperature range from room temperature to 100 °C, the nanocomposite shows a mass loss of about 5.7%. This endothermic peak corresponds to the dehydration of carrageenan and the weight loss is usually limited by the evaporation of adsorbed water from the bulk of the sample without its destruction [55]. Next, the main decomposition process occurs, which ends at 400 °C with a mass loss of about 47.8%. The DSC curve shows an intense exothermic peak at the beginning of this range with a maximum of 162 °C. Although the physicochemical processes occurring during the conversion of carrageenan to carbon are complex, the depolymerization of macromolecular chains is clearly due to the gradual oxidation of the polysaccharide matrix to low molecular weight oxygen-containing compounds [56]. After attaining this peak, an intense exothermic peak with a maximum of 305 °C is observed. This next stage of degradation may be attributed to the loss of the –OSO_3_ group from the pendant chains attached to the polymeric backbone and also may be due to the carbohydrate backbone fragmentation [57,58]. In the final stage in the range of 400–800 °C, a loss of about 24.7% of the mass occurs, which may be due to a change in the structure of manganese oxide nanocrystals, including the decomposition of inorganic salts present in *ι*-CG. Overall, these results are consistent with previous thermal behavior results for carrageenans [59,60]. The residual mass of the sample is 21.7% and represents unoxidized carbon residue with high manganese content. The changes did not significantly impact the late stages of thermal degradation in the inorganic residue, which is apparently due to the low mass content of manganese introduced into the polysaccharide.

### 3.3. Structural Features of the ι-CG-Mn Bionanocomposites

**Figure 7:**
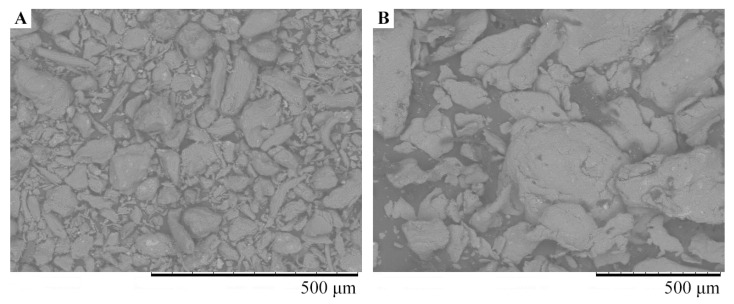
The SEM images of the original *ι*-CG (**A**) and the nanocomposite *ι*-CG-Mn (**B**).

For a comparative analysis of the supramolecular organization of the *ι*-CG-Mn and *ι*-CG, micrographs were obtained using scanning electron microscopy (SEM), see Figure 7. The nanocomposite, in comparison with the original *ι*-CG, still appears as a fine dispersion but forms larger particles than the polysaccharide alone. This bionanocomposite retains solubility; the *ι*-CG-Mn can be easily suspended in water and can also easily undergo gelation.

**Figure 8:**
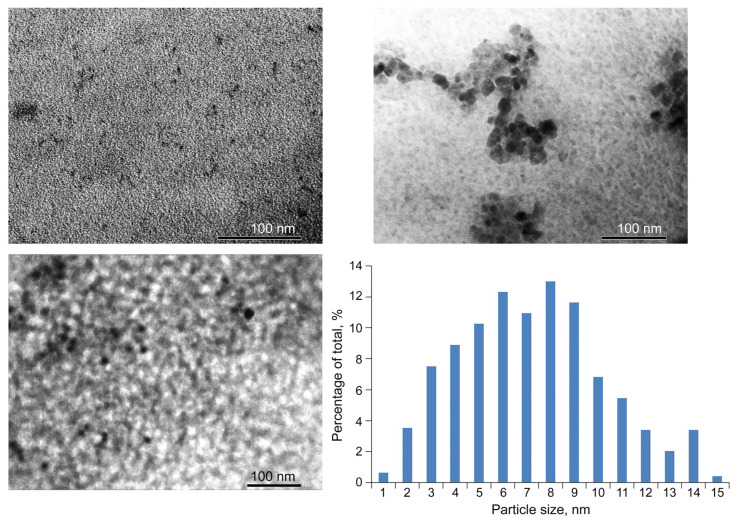
The TEM micrographs and size distribution of nanoparticles of *ι*-CG-Mn. The scale bar size is 100 nm.

Transmission electron microscopy (TEM) micrographs of the *ι*-CG-Mn nanocomposite show a distribution of dispersed nanoparticles in the form of dark, very homogeneous, predominantly spherical particles (Figure 8), in which the electron density is higher than the surrounding lighter region of the biopolymer. As can be seen, the nanoparticles are separated from each other by regions of polysaccharide molecules at a distance equal to or greater than their diameter. The sizes of manganese-containing nanoparticles in the composite range from 1 to 15 nm, but with a predominant content of nanoparticles with sizes of 3–11 nm.

### 3.4. Antibacterial Activity of ι-CG-Mn Against Cms Phytopathogen

Our final goal was to produce a multifunctional microfertilizer, but firstly we needed to evaluate the antibacterial effect of the synthesized *ι*-CG-Mn nanocomposite. Articles have already reported that metal-containing polysaccharide-based bionanocomposites are bioactive substances, including their activity for plant protection [61,62,63]. Therefore, we conducted a series of experiments to test the effect of *ι*-CG-Mn on the viability of the phytopathogen *Cms*, a Gram-positive bacterium. The infection with *Cms* is latent and manifests itself as wilt and yellowing of the stems during the growing season, and the problem is accentuated by the lack of efficient methods to combat this bacterium [22,23]. To start we needed to choose the effective concentrations of *ι*-CG-Mn. Hence, we took as a guide the concentrations used for polysaccharide-based nanocomposites in previous experiments in solution [7,12,29,30]. The concentrations correspond to the growing conditions of agricultural plants under factor-static conditions in the Murashige–Skoog nutrient medium, where manganese is added in the form of MnSO_4_ × 4H_2_O crystal hydrate [64]. In preliminary experiments, we studied the effect of polysaccharides (arabinogalactan, arabinogalactan sulfate, and *κ*-carrageenan) on potatoes (*Solanum tuberosum* L.) and bacteria, where the polysaccharide used did not have a negative effect on plants, and even stimulated the growth of the *Cms* colonies and rhizosphere bacteria [12,23,28].

**Figure 9:**
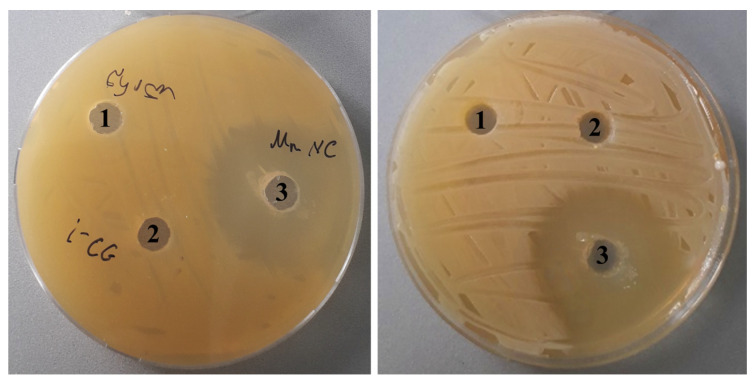
The bactericidal impact of an aqueous solution of *ι*-CG-Mn compared to the pure *ι*-CG and the control on *Cms* in agar plates; 1—control, 2—*ι*-CG, 3—*ι*-CG-Mn.

We here investigated the bactericidal activity of *ι*-CG-Mn using the well-known agar diffusion method [31], so we could observe at which concentration a dramatic decrease in the viability of the phytopathogen happened. We found that the inhibition zone of bacterial growth around *ι*-CG-Mn reached 13.0 ± 0.3 mm, whereas inhibition of bacterial growth was not observed, neither around the control (*Cms* alone) nor around the pure *ι*-CG supplemented spots (Figure 9). The pH of the agar was 7.2, at the beginning of the experiment the pH of the medium with *Cms* bacterial culture was 6.5, and at the end, after 72 h, it changed to 5.4.

**Figure 10:**
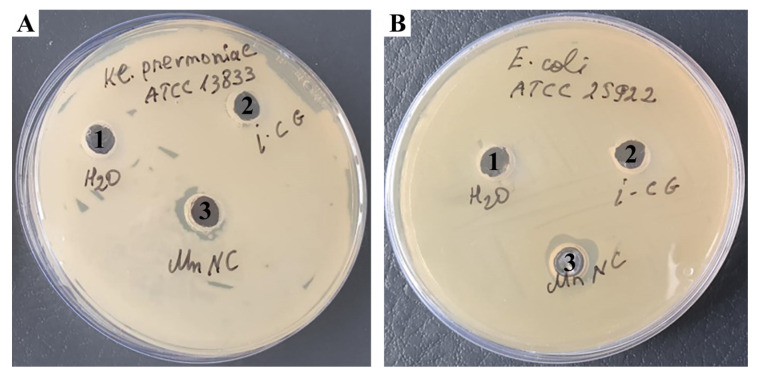
The bactericidal impact of an aqueous solution of *ι*-CG-Mn compared to the pure *ι*-CG and the control on *K. pneumoniae* (**A**) and *E. coli* (**B**) in agar plates; 1—control, 2—*ι*-CG, 3—*ι*-CG-Mn.

We also conducted experiments on two Gram-negative bacteria *Klebsiella pneumoniae* (*K. pneumoniae*) and *Escherichia coli* (*E. coli*). *K. pneumoniae* naturally occurs in the soil as a free-living diazotroph and is of agricultural interest, as it has been demonstrated to increase crop yields in agricultural conditions [65]. *E. coli* is the most widely studied prokaryotic model organism and an important species in the fields of biotechnology and microbiology [66]. As a result of the experiments, in the case of *ι*-CG-Mn, the zone of inhibition was in both cases significantly smaller than that of *Cms* (Figure 10).

**Figure 11:**
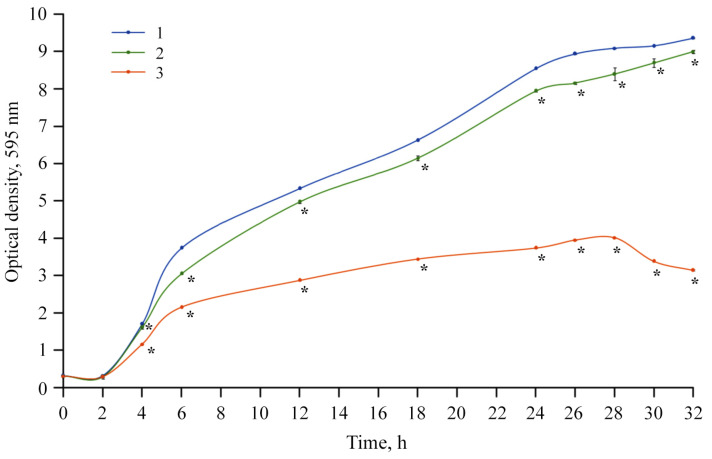
Determination of the optical turbidity (D_595_ nm) of the bacterial suspension and effect of *ι*-CG-Mn treatment in volume on growth of *Cms* pathogen; 1—control, 2—*ι*-CG, 3—*ι*-CG-Mn; * *p* ≤ 0.01 compared to the control according to the Mann–Whitney test.

Finally, we estimated the number of bacteria in a liquid medium using a method involving the optical turbidity (D_595_ nm) of the *Cms* bacterial suspension described in the literature [67]. Turbidity in samples of the control, *ι*-CG variants, and *ι*-CG-Mn treated suspensions is shown in Figure 11.

## 4. Discussion

We synthesized a nanocomposite *ι*-CG-Mn based on naturally sulfated *ι*-CG with manganese (hydr)oxide nanoparticles (Figure 3, Figure 4 and Figure 5). The nanoparticles are stabilized in the biopolymer matrix, by, e.g., coordination bonds and occupy conformational voids in the macromolecules. The biopolymer *ι*-CG itself represents a complex, but highly structured conformation as a single helix, although this does not preclude the possibility of superhelical formation or intermolecular dimeric double helices when increasing the polymer concentration [68]. The presence of a large number of sulfate groups in the polymer renders soft gel-similar properties to the final bionanocomposite. According to the FTIR and Raman spectral data, and spectral characteristics in Figure 2 and Figure 3, Mn^2+^ ions form characteristic coordination bonds with oxygen-containing groups (such as –OH, –CH_2_OH, etc.) of the polysaccharide matrix, as previously reported in the literature [12,36,69].

Our thermal stability analysis comparing *ι*-CG-Mn and pure *ι*-CG shows that the TG-DSC thermal curves for both substances have a similar decomposition pattern (Figure 6). At the same time, the thermal combustion of *ι*-CG-Mn occurs more dynamically, and the values of the temperature of decomposition in the oxidation process indicate that the combustion of the organic matrix in the presence of manganese (hydr)oxide nanoparticles begins earlier. In the first stage of the dehydration, from room temperature to 100 °C, a more pronounced endothermic effect is observed in the case of *ι*-CG-Mn, which is apparently due to additional heat absorption by metal oxide nanoparticles [70], and weight loss is due to moisture evaporation. In the second stage, the peak of active oxidation of carrageenan, which decreases from 182 °C to 162 °C for *ι*-CG compared to *ι*-CG-Mn, is especially pronounced. This effect is caused by the processes of gradual oxidation of carrageenan to low molecular weight compounds with the formation of water and carbon oxides. The thermal effect, in the region of peak 305 °C, is associated with sulfur dioxide leaving and carbohydrate backbone fragmentation [57,58]. The presence of metal-containing nanoparticles in the matrix can lead to either an increase or a decrease in the thermal stability of biopolymers, depending on the polymer used, the nature of nanoparticles, and their interaction with the macromolecule. The decrease in the thermal resistance in the resulting *ι*-CG-Mn is apparently due to the influence of magnetic manganese oxide nanoparticles on the crosslinking of the polysaccharide chains, as in the case of the presence of magnetic material *γ*-Fe_2_O_3_ in the *ι*-CG [71]. A similar decrease in thermal stability was also observed in metal-containing nanocomposites based on the natural polysaccharide arabinogalactan and humic substances [72,73]. At the third stage, after 400 °C, thermally induced processes already occur in the inorganic residue, and weight loss might occur due to the elimination of the –CO group [57]. The change in the amount of residual mass after heat treatment can be associated with the irregular amount of inorganic salts present in the *ι*-CG [58,60], as well as with the additional contribution from the contained manganese oxide nanoparticles. At the same time, the high thermal stability of the *ι*-CG-Mn bionanocomposite is maintained, which allows autoclaving at 120 °C, which is necessary when preparing a sterile nutrient medium for plants.

The nanocomposite *ι*-CG-Mn is a light-brown powder, and the SEM picture shows it to be a dispersed material (Figure 7). Supramolecularty, the composite forms a complex nanosystem in which manganese (hydr)oxide nanoparticles are stabilized by the fibers of the *ι*-CG polysaccharide matrix, that wrap around them. TEM micrographs primarily show spherical particles (electron-dense dark spots) in the biopolymer matrix, typically in sizes ranging between 3 and 11 nm (Figure 8), which are spatially separated by the matrix at distances equal to or greater than their diameter. This feature of the spatial localization of nanoparticles permits the presence of thin polysaccharide shells between them. The EPR results also confirm the formation of manganese-containing nanoparticles in the polysaccharide matrix *ι*-CG and allow the EPR method to monitor the migration and accumulation of magnetic nanoparticles and magnetic metal ions in plant tissues in further studies in the development of microfertilizers [12,16].

*ι*-CG is different from other previously studied sulfated polysaccharides (*κ*-carrageenan and sulfated arabinogalactan) [12] due to the structure of the macromolecular chain and the specific type of packing of the fiber [18,74]. *κ*-Carrageenan is less ionized, with one sulfate group per two galactose molecules, and forms more strong and rigid gels [18,20], while arabinogalactan is an intertwined branched helical system with greater water solubility due to its hydrodynamic properties [34,74,75]. In turn, these differences affect the amount of manganese accumulated in the biopolymer, as well as the size and distribution of manganese (hydr)oxide nanoparticles. We conclude that better solubility at lower temperatures and the formation of more elastic gels enable the creation of smaller and more uniformly stabilized metal-containing nanoparticles in the macromolecule mass and provide the bionanocomposite with greater possibilities to create a nutrient medium for plant material.

In the course of this research, we also studied the bacteriostatic properties of the resulting bionanocomposite *ι*-CG-Mn that are essential for the development of future microfertilizers. A series of publications have demonstrated the success of using manganese oxide nanoparticles as antibacterial agents [76,77,78]. Based on the results of our experiments, *ι*-CG-Mn has a pronounced antibacterial effect on the phytopathogenic bacterium *Cms*. As seen in Figure 9 and Figure 11, the concentration of *Cms* in the biological suspension treated with *ι*-CG-Mn decreased by approximately three times compared to the control. The addition of the original *ι*-CG had no significant impact on the increase in biomass in comparison with the control. The duration of the test was 34 h, and the exponential phase was prolonged in both variants for about 24 h. The decline under the influence of the *ι*-CG-Mn was slow, but the rate of decline increased after 28 h. A study of the impact of the nanocomposite on liquid bacterial culture revealed that the nanocomposite *ι*-CG-Mn inhibited the growth of the *Cms* strain for the most part. The results correspond to approximately 67% of the control. This reduced growth may be due to the disruption of the cell membrane potential with subsequent destruction of the membrane caused by nanoparticles attaching to its surface, as has been shown for *Cms* using, e.g., selenium-containing nanocomposites [79]. Similar effects have been reported for manganese-containing nanocomposites based on polysaccharides [12], where the most pronounced effect was found in sulfated polysaccharides. Consequently, we have chosen the more sulfated *ι*-CG for this study as particularly interesting for the development of multifunctional microfertilizers. Indeed, in comparison with the manganese-containing nanocomposites based on *κ*-carrageenan tested in previous works [12], the *ι*-CG-Mn examined here shows a more marked antibacterial effect similar to the nanocomposites based on synthetically sulfated arabinogalactan. However, arabinogalactan is a much more expensive polysaccharide.

In relation to *K. pneumoniae* and *E. coli*, the *ι*-CG-Mn did not show any pronounced bactericidal activity, which can be explained by the structure of the bacterial membrane of these cultures. Since these cultures are Gram-negative bacteria, they have a thin layer of peptidoglycan, and an outer membrane made of lipopolysaccharides. The outer membrane acts as a barrier, preventing antimicrobial agents such as nanoparticles from penetrating. *Cms* is a Gram-positive bacterium with a thicker peptidoglycan layer, but *Cms* does not have an outer membrane, making it more susceptible to the action of nanoparticles that can interact more effectively with the cell wall. The outer membrane of Gram-negative bacteria may limit the capacity of *ι*-CG-Mn to penetrate the cell and exert their antimicrobial activity, resulting in a reduced inhibition zone for *K. pneumoniae* and *E. coli* [80].

Our results confirm that the synthesized bionanocomposite can act as a carrier of microfertilizer metal ions to stimulate the development of agricultural plants, and simultaneously be an effective agent against the phytopathogen *Cms*. It is very important to add that we have previously shown [12,23,28] that polysaccharides have no negative effect on plant growth, and composites based on these polysaccharides stimulate the development of the plants [12,21]. In sum, using *ι*-CG-Mn seems promising, since the *ι*-CG polysaccharide is widely available and significantly cheaper than some other biopolymers. Moreover, *ι*-CG-Mn does not require the synthetic introduction of a sulfate group into the polysaccharide structure as is common for arabinogalactan, cellulose, pectin, etc.

## 5. Conclusions

We synthesized a bionanocomposite *ι*-CG-Mn based on the natural polysaccharide *ι*-CG with manganese (hydr)oxide nanoparticles in the range of 3–11 nm embedded in the biopolymer matrix. We demonstrate the promising and advantageous properties of this composite as a basis for the research of novel safe, biodegradable, and non-toxic multifunctional microfertilizers (with a manganese content of 0.7–1.5 wt%) as carriers of mineral microelements for agricultural plants. Among the most important results, the *ι*-CG-Mn obtained has an antibacterial effect on the phytopathogenic bacterium *Cms*, and the nanocomposite inhibits the growth of *Cms* by 67% compared to the control. The observed activity can be explained by the synergistic effect of the bioactivity of the selected sulfated polysaccharide *ι*-CG and the antibacterial activity of the metal oxide nanoparticles. The next development stage involves research on plants in aseptic laboratory conditions, as well as in open or protected soil conditions, with the aim of developing solutions for challenges in agriculture. Moreover, the bionanocomposites obtained can also be used to grow plants on artificial nutrient media with a balanced composition of the nutrient components necessary for the growth and hence enhance the development of plants, which cultivation is difficult under normal conditions. The use of such artificial nutrient media is of great importance for the design of autonomous life support systems, for example for long-term space flights.

## Data Availability

The original contributions presented in this study are included in the article. Further inquiries can be directed to the corresponding author.

## References

[1] Ochsner A., Shokuhfar A. (2013). New Frontiers of Nanoparticles and Nanocomposite Materials. Novel Principles and Techniques.

[2] Ben Amor I., Hemmami H., Grara N., Aidat O., Ben Amor A., Zeghoud S., Bellucci S. (2024). Chitosan: A green approach to metallic nanoparticle/nanocomposite synthesis and applications. Polymers.

[3] Pozdnyakov A.S., Emel’yanov A.I., Kuznetsova N.P., Ermakova T.G., Korzhova S.A., Khutsishvili S.S., Vakul’skaya T.I., Prozorova G.F. (2019). Synthesis and characterization of silver containing nanocomposites based on 1-vinyl-1,2,4-triazole and acrylonitrile copolymer. J. Nanomater..

[4] Rozenberg B.A., Tenne R. (2008). Polymer-assisted fabrication of nanoparticles and nanocomposites. Prog. Polym. Sci..

[5] Wang C., Gao X., Chen Z., Chen Y., Chen H. (2017). Preparation, characterization and application of polysaccharide-based metallic nanoparticles: A review. Polymers.

[6] Mavelil-Sam R., Ouseph E.M., Morreale M., Scaffaro R., Thomas S. (2023). Recent developments and formulations for hydrophobic modification of carrageenan bionanocomposites. Polymers.

[7] Perfileva A.I., Nozhkina O.A., Ganenko T.V., Graskova I.A., Sukhov B.G., Artem’ev A.V., Trofimov B.A., Krutovsky K.V. (2021). Selenium nanocomposites in natural matrices as potato recovery agent. Int. J. Mol. Sci..

[8] Nandini B., Mawale K.S., Giridhar P. (2023). Nanomaterials in agriculture for plant health and food safety: A comprehensive review on the current state of agro-nanoscience. 3 Biotech.

[9] Pan D., Schmieder A.H., Wickline S.A., Lanza G.M. (2011). Manganese-based MRI contrast agents: Past, present and future. Tetrahedron.

[10] Mahlangeni N.T., Moodley R. (2021). Biosynthesis of manganese oxide nanoparticles using *Urginea sanguinea* and their effects on cytotoxicity and antioxidant activity. Adv. Nat. Sci. Nanosci. Nanotechnol..

[11] Bravo A., Anacona J.R. (2001). Metal complexes of the flavonoid quercetin: Antibacterial properties. Transit. Met. Chem..

[12] Khutsishvili S.S., Perfileva A.I., Nozhkina O.A., Ganenko T.V., Krutovsky K.V. (2021). Novel nanobiocomposites based on natural polysaccharides as universal trophic low-dose micronutrients. Int. J. Mol. Sci..

[13] Burnell J.N., Graham R.D., Hannam R.J., Uren N.C. (1988). The biochemistry of manganese in plants. Manganese in Soils and Plants. Developments in Plant and Soil Sciences.

[14] Schmidt S.B., Jensen P.E., Husted S. (2016). Manganese deficiency in plants: The impact on photosystem II. Trends Plant Sci..

[15] Wallace T. (1943). The diagnosis of mineral deficiencies in plants by visual symptoms a colour atlas and guide. Nature.

[16] Khutsishvili S.S., Perfileva A.I., Nozhkina O.A., Dyrkach A.Y. (2022). EPR Study of accumulation and toxic effect of iron and copper during the development of *Solanum tuberosum* L. in vitro. J. Appl. Spectr..

[17] Trono G.C., Lluisma A.O. (1992). Differences in biomass production and carrageenan yields among four strains of farmed carrageenophytes in Northern Bohol, Philippines. Hydrobiologia.

[18] Campo V.L., Kawano D.F., da Silva D.B., Carvalho I. (2009). Carrageenans: Biological properties, chemical modifications and structural analysis—A review. Carbohydr. Polym..

[19] Toumi S., Yahoum M.M., Lefnaoui S., Hadjsadok A., Sid A.N.E.H., Hassein-Bey A.H., Amrane A., Zhang J., Assadi A.A., Mouni L. (2023). Development of new alkylated carrageenan derivatives: Physicochemical, rheological, and emulsification properties assessment. Sustainability.

[20] Tuvikene R., Phillips G.O., Williams P.A. (2020). Carrageenans. Handbook of Hydrocolloids.

[21] Khutsishvili S.S., Perfileva A.I., Kon’kova T.V., Lobanova N.A., Sadykov E.K., Sukhov B.G. (2024). Copper-containing bionanocomposites based on natural raw arabinogalactan as effective vegetation stimulators and agents against phytopathogens. Polymers.

[22] Eichenlaub R., Gartemann K.-H. (2011). The *Clavibacter michiganensis subspecies*: Molecular investigation of gramm-positive bacterial plant pathogens. Annu. Rev. Phytopathol..

[23] Li X., Tambong J., Yuan K.X., Chen W., Xu H., Lévesque C.A., De Boer S.H. (2018). Re-classification of *Clavibacter michiganensis subspecies* on the basis of whole-genome and multi-locus sequence analyses. Int. J. Syst. Evol. Microbiol..

[24] Khutsishvili S.S., Ganenko T.V., Sukhov B.G. (2021). Formation and paramagnetic properties of manganese-containing bionanocomposites based on natural polysaccharide matrices. J. Carbohydr. Chem..

[25] Irawan V., Masaki T., Toshiyuki I. (2020). Apatite coating of iron oxide nanoparticles by alternate addition of calcium and phosphate solutions: A calcium and carboxylate (Ca-COO) complex-mediated apatite deposition. J. Inorg. Organomet. Polym..

[26] Sadunishvili T., Węgierek-Maciejewska A., Arseniuk E., Gaganidze D., Amashukeli N., Sturua N., Amiranashvili L., Kharadze S., Kvesitadzeet G. (2020). Molecular, morphological and pathogenic characterization of *Clavibacter michiganensis* subsp. *sepedonicus* strains of different geographic origins in Georgia. Eur. J. Plant Pathol..

[27] Roozen N.J.M., Van Vuurde J.W.L. (1991). Development of a semi-selective medium and an immunofluorescence colony-staining procedure for the detection of *Clavibacter michiganensis* subsp. *sepedonicus* in cattle manure slurry. Neth. J. Plant Pathol..

[28] Perfileva A.I., Nozhkina O.A., Graskova I.A., Sukhov B.G., Trofimov B.A. (2020). Carrageenan as polymer matrix for selenium nanocomposites. Russ. Chem. Bull..

[29] Perfileva A.I., Nozhkina O.A., Graskova I.A., Sidorov A.V., Lesnichaya M.V., Aleksandrova G.P., Dolmaa G., Klimenkov I.V., Sukhov B.G. (2018). Synthesis of selenium and silver nanobiocomposites and their influence on phytopathogenic bacterium *Clavibacter michiganensis* subsp. *sepedonicus*. Russ. Chem. Bull..

[30] Perfileva A.I., Tsivileva O.M., Nozhkina O.A., Karepova M.S., Graskova I.A., Ganenko T.V., Sukhov B.G., Krutovsky K.V. (2021). Effect of natural polysaccharide matrix-based selenium nanocomposites on *Phytophthora cactorum* and rhizospheric microorganisms. Nanomaterials.

[31] Sagdic O., Aksoy A., Ozkan G. (2006). Evaluation of the antibacterial and antioxidant potentials of gilaburu (*Viburnum opulus* L.) fruit extract. Acta Aliment..

[32] Dhital S., Amatya S.P., Aryal S., Neupane P., Parajuli N., Tamang M., Thanait P. (2024). Synthesis of manganese oxide nanoparticles using co-precipitation method and its antimicrobial activity. Int. J. New. Chem..

[33] Lesnichaya M.V., Sukhov B.G., Aleksandrova G.P., Gasilova E.R., Vakul’skaya T.I., Khutsishvili S.S., Sapozhnikov A.N., Klimenkov I.V., Trofimov B.A. (2017). Chiroplasmonic magnetic gold nanocomposites produced by one-step aqueous method using κ-carrageenan. Carbohydr. Polym..

[34] Khutsishvili S.S., Vakul’skaya T.I., Aleksandrova G.P., Sukhov B.G. (2017). Strong stabilization properties of humic substance matrixes for silver bionanocomposites. Micro Nano Lett..

[35] Zaafarany I., Gobouri A., Hassan R. (2013). Oxidation of some sulfated carbohydrates: Kinetics and mechanism of oxidation of chondroitin-4-sulfate by alkaline permanganate with novel synthesis of coordination biopolymer precursor. J. Mater. Sci. Res..

[36] Kusumaningrum R., Widayatno W.B., Wismogroho A.S., Nugroho D.W., Rochman N.T., Amal M.I., Noviyanto A. (2019). Reactivity of manganese sulphate from Sumbawa manganese ore with precipitating agent: Theoretical and experimental evaluation. J. Phys. Conf. Ser..

[37] Chitra R., Sathya P., Selvasekarapandian S., Monisha S., Moniha V., Meyvel S. (2019). Synthesis and characterization of iota-carrageenan solid biopolymer electrolytes for electrochemical applications. Ionics.

[38] Tajmir-Riahi H.A. (1989). Carbohydrate metal ion complexes. Interaction of D-glucono-1,5-lactone with Zn(II), Cd(II), and Hg(II) ions in the solid and aqueous solution, studied by ^13^C-NMR, FT-IR, and X-ray powder diffraction measurements. Can. J. Chem..

[39] De Souza R.F.V., De Giovani W.F. (2005). Synthesis, spectral and electrochemical properties of Al(III) and Zn(II) complexes with flavonoids. Spectrochim. Acta A.

[40] Nikolić G.S., Cakić M.D., Nikolić G.S. (2011). Analysis of bioactive oligosaccharide-metal complexes by modern FTIR spectroscopy: Copper complexes. Fourier Transforms—New Analytical Aproaches and FTIR Strategies.

[41] Morsy M., Gomaa I., Abd Elhamid A.E.M., Shawkey H., Aly M.A.S., Elzwawy A. (2023). Ternary nanocomposite comprising MnO_2_, GQDs, and PANI as a potential structure for humidity sensing applications. Sci. Rep..

[42] Lis T., Matuszewski J. (1979). Manganese(II) malonate dihydrate: A reinvestigation. ActaCryst. B.

[43] Pereira L., Amado A.M., Critchley A.T., Van de Velde F., Ribeiro-Claro P.J. (2009). Identification of selected seaweed polysaccharides (phycocolloids) by vibrational spectroscopy (FTIR-ATR and FT-Raman). Food Hydrocoll..

[44] Mahardika A., Susanto A.B., Pramesti R., Matsuyoshi H., Andriana B.B., Matsuda Y., Sato H. (2019). Application of imaging Raman spectroscopy to study the distribution of *Kappa* carrageenan in the seaweed *Kappaphycus alvarezii*. J. Appl. Phycol..

[45] Liu Y., Shi Y., Cai L., Hao Y., Zhao C. (2013). Determination of copper, zinc, cadmium and lead in water using co-precipitation method and Raman spectroscopy. J. Innov. Opt. Health Sci..

[46] Zhang J., Li Y., Wang L., Zhang C., He H. (2015). Catalytic oxidation of formaldehyde over manganese oxides with different crystal structures. Catal. Sci. Technol..

[47] Souri M., Hoseinpour V., Ghaemi N., Shakeri A. (2019). Procedure optimization for green synthesis of manganese dioxide nanoparticles by *Yucca gloriosa* leaf extract. Int. Nano Lett..

[48] Corrales J., Acosta J., Castro S., Riascos H., Serna-Galvis E., Torres-Palma R.A., Ávila-Torres Y. (2022). Manganese dioxide nanoparticles prepared by laser ablation as materials with interesting electronic, electrochemical, and disinfecting properties in both colloidal suspensions and deposited on fluorine-doped tin oxide. Nanomaterials.

[49] Mozaffari H., Mahdieh M.H. (2019). Enhancement of ablation rate and production of colloidal nanoparticles by irradiation of metals with nanosecond pulsed laser in presence of external electric field. Phys. Lett. A.

[50] Ingram D.J.E. (1969). Biologycal and Biochemical Applications of Electron Spin Resonance.

[51] Möncke D., Ehrt D., Kamitsos E.I. (2013). Spectroscopic study of manganese-containing borate and borosilicate glasses: Cluster formation and phase separation. Phys. Chem. Glasses B.

[52] Galyametdinov Y.G., Sagdeev D.O., Sukhanov A.A., Voronkova V.K., Shamilov R.R. (2019). Monitoring of the mechanism of Mn ions incorporation into quantum dots by optical and EPR spectroscopy. Photonics.

[53] Anderson P.W., Weiss P.R. (1953). Exchange narrowing in paramagnetic resonance. Rev. Mod. Phys..

[54] Elsi S., Pushpanathan K. (2019). Role of Cu and Mn dopants on *d*^0^ ferromagnetism of ZnS nanoparticles. J. Mater. Sci. Mater..

[55] Dumanlı A.G., Windle A.H. (2012). Carbon fibres from cellulosic precursors: A review. J Mater Sci..

[56] Huang X. (2009). Fabrication and properties of carbon fibers. Materials.

[57] Mishra D.K., Tripathy J., Behari K. (2008). Synthesis of graft copolymer (κ-carrageenan-g-N,N-dimethylacrylamide) and studies of metal ion uptake, swelling capacity and flocculation properties. Carbohydr. Polym..

[58] Freile-Pelegrin Y., Azamar J.A., Robledo D. (2011). Preliminary characterization of carrageenan from the red seaweed *Halymenia floresii*. J. Aqua. Food Prod. Technol..

[59] Ma S., Chen L., Liu X., Li D., Ye N., Wang L. (2012). Thermal behaviour of carrageenan: Kinetic and characteristic studies. Int. J. Green Energy.

[60] Mahmood W.A.K., Khan M.M.R., Teow T.C. (2014). Effects of reaction temperature on the synthesis and thermal properties of carrageenan ester. J. Phys. Sci..

[61] Huang Q., Jin Y., Zhang L., Cheung P.C.K., Kennedy J.F. (2007). Structure, molecular size and antitumor activities of polysaccharides from Poria cocos mycelia produced in fermenter. Carbohydr. Polym..

[62] Tong H., Xia F., Feng K., Sun G., Gao X., Sun L., Jiang R., Tian D., Sun X. (2009). Structural characterization and in vitro antitumor activity of a novel polysaccharide isolated from the fruiting bodies of *Pleurotus ostreatus*. Bioresour. Technol..

[63] Moradali M.-F., Mostafavi H., Ghods S., Hedjaroude G.-A. (2007). Immunomodulating and anticancer agents in the realm of macromycetes fungi (macrofungi). Int. Immunopharm..

[64] Murashige T., Skoog F. (1962). A received medium for rapid growth and bio assays with tobacco tissue culture. Plant Physiol..

[65] Riggs P.J., Chelius M.K., Iniguez A.L., Kaeppler S.M., Triplett E.W. (2001). Enhanced maize productivity by inoculation with diazotrophic bacteria. Aust. J. Plant Physiol..

[66] Tenaillon O., Skurnik D., Picard B., Denamur E. (2010). The population genetics of commensal Escherichia coli. Nat. Rev. Microbiol..

[67] Lesnichaya M., Perfileva A., Nozhkina O., Gazizova A., Graskova I. (2022). Synthesis, toxicity evaluation and determination of possible mechanisms of antimicrobial effect of arabinogalactane-capped selenium nanoparticles. J. Trace Elem. Med. Biol..

[68] Schefer L., Adamcik J., Mezzenga R. (2014). Unravelling secondary structure changes on individual anionic polysaccharide chains by atomic force microscopy. Angew. Chem. Int. Ed..

[69] Rendleman J.A. (1978). Metal-polysaccharide complexes—Part I. Food Chem..

[70] Falsafi S.R., Topuz F., Bajer D., Mohebi Z., Shafieiuon M., Heydari H., Rawal S., Sathiyaseelan A., Wang M.-H., Khursheed R. (2023). Metal nanoparticles and carbohydrate polymers team up to improve biomedical outcomes. Biomed. Pharmacother..

[71] Maciel D.J., Ferreira I.L.M., da Costa G.M., da Silva M.R. (2016). Nanocomposite hydrogels based on iota-carrageenan and maghemite: Morphological, thermal and magnetic properties. Eur. Polym. J..

[72] Aleksandrova G.P., Prozorova G.F., Klimenkov I.V., Sukhov B.G., Trofimov B.A. (2016). Effect of metal nanoparticles on the thermal stability and conductivity of nanocomposites. Bull. Russ. Acad. Sci. Phys..

[73] Khutsishvili S.S., Tikhonov N.I., Pavlov D.V., Vakul’skaya T.I., Penzik M.V., Kozlov A.N., Lesnichaya M.V., Aleksandrova G.P., Sukhov B.G. (2019). Gold- and silver-containing bionanocomposites based on humic substances extracted from coals: A thermal analysis study. J. Therm. Anal. Calorim..

[74] Medvedeva A.S., Safronova L.P., Ganenko T.V., Sukhov B.G., Larina L.I., Kon’shina T.M., Kotegov V.P. (2014). Synthesis of water-soluble bioconjugate piroxicam-arabinogalactan sulfate. Russ. Chem. Bull..

[75] Ganenko T.V., Tantsyrev A.P., Sapozhnikov A.N., Khutsishvili S.S., Vakul’skaya T.I., Fadeeva T.V., Sukhov B.G., Trofimov B.A. (2015). Nanocomposites of silver with arabinogalactan sulfate: Preparation, structure, and antimicrobial activity. Russ. J. Gen. Chem..

[76] Rónavári A., Ochirkhuyag A., Igaz N., Szerencsés B., Ballai G., Huliák I., Bocz C., Kovács A., Pfeiffer I., Kiricsi M. (2024). Preparation, characterization and in vitro evaluation of the antimicrobial and antitumor activity of MnOx nanoparticles. Colloids Surf. A Physicochem. Eng. Asp..

[77] Saod W.M., Hamid L.L., Alaallah N.J., Ramizy A. (2022). Biosynthesis and antibacterial activity of manganese oxide nanoparticles prepared by green tea extract. Biotechnol. Rep..

[78] Du T., Chen S., Zhang J., Li T., Li P., Liu J., Du X., Wang S. (2020). Antibacterial activity of manganese dioxide nanosheets by ROS-mediated pathways and destroying membrane integrity. Nanomaterials.

[79] Perfileva A.I., Moty’leva S.M., Klimenkov I.V., Arsent’ev K.Y., Graskova A.I., Sukhov B.G., Trofimov B.A. (2017). Development of antimicrobial nano-selenium biocomposite for protecting potatoes from bacterial phytopathogens. Nanotechnol. Russ..

[80] Breijyeh Z., Jubeh B., Karaman R. (2020). Resistance of gram-negative bacteria to current antibacterial agents and approaches to resolve it. Molecules.

